# Improving the sense of city belonging among migrant elderly following family from an elderly service perspective: a cross-sectional study

**DOI:** 10.1186/s12889-022-14445-6

**Published:** 2022-11-07

**Authors:** Meijie Chu, Chun-Yang Lee, Lamu Suona, Min Gao, Tianmu Chen, Shuoxun Zhang, Yi-Chen Chiang

**Affiliations:** 1 State Key Laboratory of Molecular Vaccinology and Molecular Diagnostics, School of Public Health, Xiamen University, Xiang’an South Road, Xiang’an District 361102 Xiamen, China; 2 School of International Business, Xiamen University Tan Kah Kee College, Zhangzhou, China; 3 Lasa Customs District, P.R. China, Lasa, China; 4 Business School, Sichuan University, No. 29 Wangjiang Road, Wuhou District, Sichuan 610064 Chengdu, China

**Keywords:** Migrant elderly following family, Sense of city belonging, Elderly service, Appropriate strategy, With or without hypertension/diabetes

## Abstract

**Background:**

The migrant elderly following family (MEFF), who migrates to new community to reunite with families, may face challenges of city integration and belonging. This study aims to explore from an elderly service perspective how to improve the sense of city belonging for MEFFs with and without hypertension/diabetes conditions.

**Methods:**

Data were derived from the 2017 China Migrants Dynamic Survey and China National Statistical Yearbooks in 2017. The study included 882 MEFFs with hypertension or diabetes and 1266 MEFFs without hypertension and diabetes. Hierarchical linear modeling was applied to analyze the effects of individual and provincial elderly services on sense of city belonging among the MEFF with and without hypertension/diabetes.

**Results:**

The MEFFs with hypertension or diabetes exhibited a greater sense of city belonging when they were familiar with a wider range of health education topics (γ = 0.05, *p* = 0.033) and were in those provinces with a greater number of licensed doctors (γ = 0.39, *p* < 0.001) and hospitals (*p* = 0.042). For those MEFFs without hypertension or diabetes, social security cards (γ = 0.57, *p* < 0.001) and awareness of a wider range of health education topics (γ = 0.07, *p* = 0.018) may help to improve their sense of city belonging.

**Conclusion:**

This study calls for strengthening the accessibility in inclusive elderly services, and minimizing or even eliminating the inequality in elderly services at the individual and provincial levels to increase sense of city belonging among the MEFFs. For the MEFFs with hypertension or diabetes, health managers should focus on improving health information dissemination and increasing the number of doctors per 1000 people as well as and the number of hospitals to enhance the sense of city belonging. Moreover, the government should strengthen social security and health education to facilitate the adaptation and integration of MEFFs without hypertension and diabetes into the host city.

## Background

With the transformation of China’s economy and the acceleration of the aging population, the scale of Chinese elderly migrants has been constantly expanding. The number of internal migrants who migrate between regions in one country has increased from 221.43 million in 2010 to 375.82 million in 2020, with an average annual growth rate of 6.97% in China [1]. The number of migrants aged 60 and older reached 18.0 million in 2015, which accounts for 7.2% of all internal migrants [2]. Most elderly migrants migrate from their original residences to new places to reunite with their families and take care of the younger generations [3]. This group of people is referred to as the elderly migrant following family (MEFF) [4, 5]. Skip-generation raising is beneficial for reducing the negative effects of fertility on young people’s work and, in turn, brings value to society. However, the MEFF lives in the host city as part of the nonlocal (county, city) hukou system. Hence, in contrast to the local residents, most migrants are less likely to have access to health services [6–9], and many of the elderly migrants may face numerous life challenges in urban areas [8]. Due to the different living backgrounds and environments, the shrinking/weakening of family/friendship ties, social capital reconstruction, and the lack of available social resources, the MEFFs must contend with integrating into a new community and being accepted by the local elderly, especially given that feelings of belonging, access to social resources, and quality health welfare are important facets for the elderly [10]. That said, as most migrants struggle to attain a sense of belonging in their new environments, studies and policies aimed to promote healthy aging should focus specifically on the needs of elderly services and improve the sense of belonging among the MEFF population.

### Sense of belonging

The term sense of belonging is defined as “the experience of personal involvement in a system or environment so that persons feel themselves to be an integral part of that system or environment” [11]. Belongingness refers to a human emotional need to develop internal relationships and be accepted by the members of one’s social circle [12, 13]. Research indicates that the confusion between a sense of belonging and one’s identity is associated with psychological pain among middle-aged and elderly migrants [14]. Research indicates that the confusion between a sense of belonging and one’s identity is associated with psychological pain among middle-aged and elderly migrants [14]. Migrants with a strong sense of belonging to the host city and to their original residence have fewer mental health problems than those who had a strong sense of belonging to their original residence but not to their host city [15]. Across a range of populations, sense of belonging and group membership has been shown to be associated with psychological well-being [16]. Thus, to positively cope with the aging population, it is critical for new urban areas to develop appropriate strategies for improving the sense of city belonging among these MEFFs.

### Influencing factors of sense of city belonging

Many studies have focused on the influencing factors of sense of city belonging. Individual socioeconomic characteristic such as age, occupation, income and longer time in the host cities could determine sense of belonging [17, 18]. Some researchers have suggested that migrants with greater social adaptation skills, language skills, interpersonal skills, connections with local residents, degrees of acceptance, and roles consciousness may exhibit a higher sense of city belonging and identity [19–23]. With respect to individual psychological feelings, some scholars indicated that life satisfaction was positively and directly correlated to immigrants’ sense of belonging [24]. The uncertainty and exclusion from the formal economy could lead to low sense of belonging [25]. Residential satisfaction would positively affect sense of belonging among older adults in the community [26]. In terms of environment and resources, good neighbourhood environment and large environment resources could contribute to sense of belonging [27]. In addition, social services could contribute to integrate migrants in their new communities [28]. Previous studies in several countries also strengthened the government and migrant service agencies could introduce public policies or supporting programs aimed at improving residential settings, social participation and decreasing discrimination to favor the sense of belonging of migrants [29, 30]. All in all, previous studies considered the determines of sense of belonging from the perspective of demographic characteristics, personal skills, psychology, surrounding environment, and social resources.

While, migrants’ health status and access to healthcare is heterogeneous across regions. A [30] study conducted in the UK suggested that it could be beneficial to increase sense of belonging for vulnerable groups by using a public health approach [31]. Migrants’ self-rated health was negatively associated with their social isolation [32]. As healthcare access and utilization is likely associated with health status [33], some studies have analyzed sense of belonging from the perspective of health-related services and concluded community services, including healthcare services and healthcare system, are associated with a sense of belonging and identity integration [34–36]. A regional analysis in Canada aimed to identify the effect of major health, health services, socio-economic status and geographic on sense of belonging. A analysis of 17 European countries found that sense of belong varied across types of welfare state regimes [37, 38]. These above results about the relationships between health resources and sense of belonging are not focused on older migrants. With the increasing number of older migrants, health and medical care are identified as priorities that deserve greater attention [38]. The relationships between elder care resources and sense of city belonging among the elder migrants and the MEFFs was needed to be further explored.

### Elderly services

Accordingly, to actively respond to population aging and create an aging-friendly social environment, there should be a focus on social security, pension, medical care and other quality of life issues for the elderly given that elderly migrants may be at high risk of health-related problems and their physiological functions deteriorate with age [39]. While elderly migrants and their families may pay more attention to elderly services and health-related resource access, for the sustainable development of society, it is necessary to focus on the improvement of elderly services and resource allocation among the MEFFs to enhance their sense of belonging. That said, the elderly services system should include not only medical aspects but also other measures, such as cultural measures, social health communication strategies, health insurance options, and training and retraining of medical staff [40, 41]. As the social security system and medical support systems are important facets of elderly services, promoting community health education also plays a significant role in disease prevention and reducing the national burden. Moreover, improving the MEFF population’s health literacy may mitigate health inequalities between local residents and migrants [42]. In addition, cultivating and building a team of health professionals are the keys to promoting the development of high quality elder care [41].

In terms of the benefit of community elder services, one study in Barcelona indicated that the community-based health intervention for the elder could help to increase their health literacy and give them a sense of belonging to a community [43]. Some elder migrants even visit neighbourhood facilities for the same language and cultural contexts of the caregivers to reduce psychological isolation [44]. To our knowledge, there are few studies focused on the MEFFs and explored the effect of elder care resources on their sense of belonging. This study presents a comprehensive strategy including the aforementioned aspects of elder services and explores ways to establish a city where the MEFFs experience a sense of belonging.

Based on Andersen’s Behavioral Model of Health Service Use, it was concluded that health status (e.g., diagnosed diseases) is an existing factor that may influence the utilization of healthcare services [45]. Similarly, patients with chronic diseases may have a lower quality of life and have inadequate social and role adaptation skills compared to individuals without chronic illnesses [46]. In addition, chronic diseases often lead to psychological disorders and mental illness [47]. Elder migrants with type 2 diabetes identified a need for greater community engagement and education [48]. Hence, this study analyzed an appropriate strategy for improving the sense of belonging among the MEFFs with hypertension/diabetes and those without hypertension/diabetes from the perspective of services for the elder.

### The present study

The combination of longevity and low fertility rates poses even greater challenges in China and other countries. To actively cope with the aging population, it is necessary to adopt policies and foster an aging-friendly social environment in which senior citizens are respected, cared for, and allowed to enjoy their later years. The existed evidence showed that there are widening health differentials between migrants and local residents with age, and informal barriers to quality healthcare for aging migrants [49]. The extant evidence fails to adequately account for the potential effects of elderly services at the individual and provincial levels with respect to the sense of city belonging among the MEFF population. Therefore, this study aims to (1) understand the status of elderly service utilization among MEFFs with and without hypertension or diabetes conditions and (2) explore how to improve the sense of city belonging among MEFFs with and without hypertension or diabetes conditions from the perspective of elderly services.

## Methods

### Participations and setting

The individual data were derived from the 2017 China Migrants Dynamic Survey (CMDS), which was conducted by the National Health Commission of the People’s Republic of China. The CMDS includes 31 provinces (autonomous regions and municipalities) in mainland China. The study employed a stratified three-stage probability proportional to size (PPS) sampling method using the annual national data on internal migrants from each province in the previous year for its basic sampling frame. The survey was representative of residents of major cities and sought a balance among key liaison cities while maintaining representation at the country and province levels.

The research sample was comprised of migrants aged 60 years and older who migrated from their original residences with their families. They had lived in the inflow area for more than one month within the nonlocal (county, city) hukou. The reasons for their migration included taking care of the younger generations, providing care for the elderly, and simply migrating with their families. In total, 2148 elderly migrants who migrated with their families were included in the analysis.

The provincial level used was obtained from the 2017 China National Statistical Yearbook, which was conducted by the Department of Population and Employment Statistics of the National Bureau of Statistics of China, covers approximately one per one-thousand of the total population of the country in 2017. As the sample survey on population change considers the whole nation as the population and each province, autonomous region and municipality as subpopulations, the stratified multi-stage systematic PPS cluster sampling scheme is used.

### Measures

**Fig. 1:**
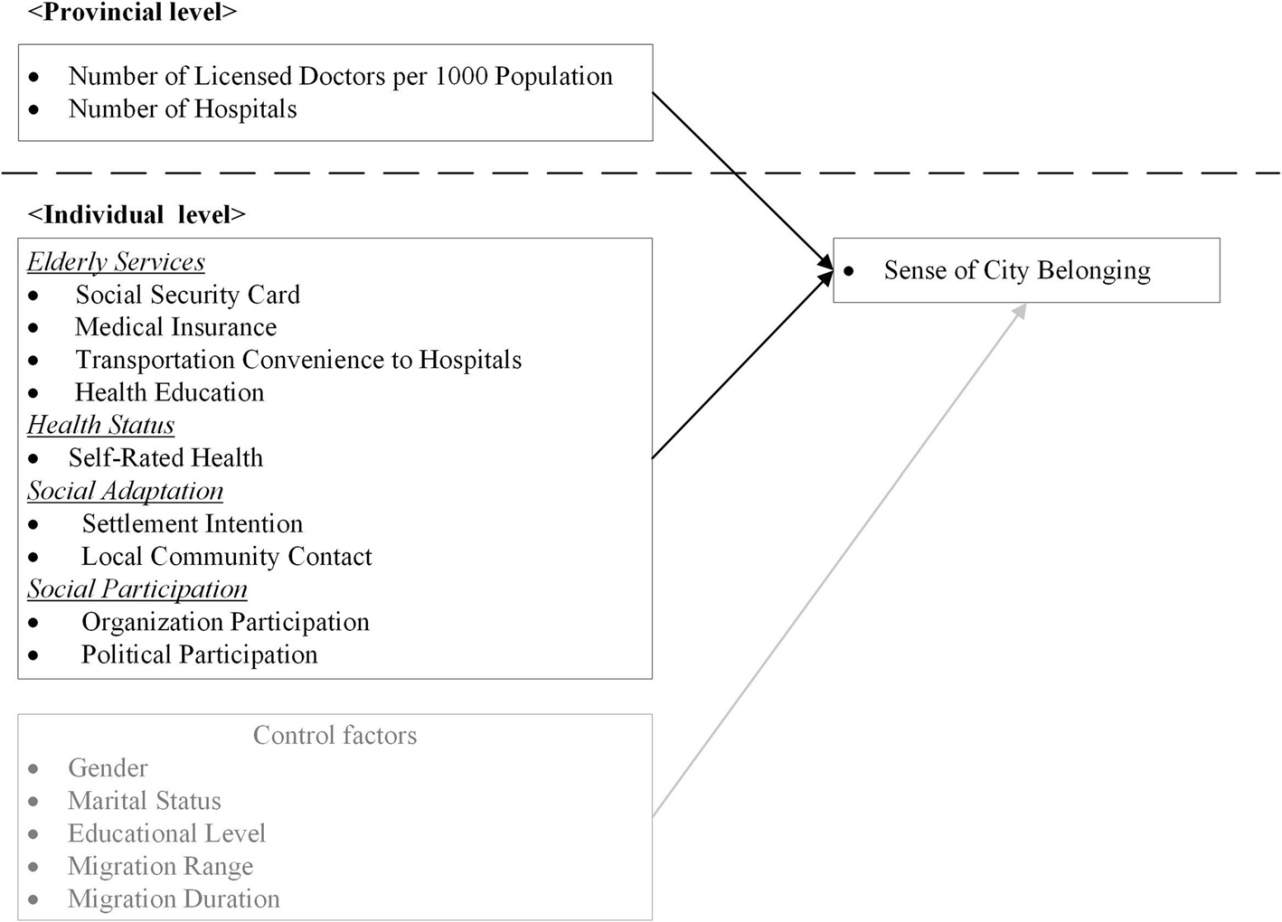
Research framework

In this analysis, the outcome of interest is the individual’s sense of city belonging. The research framework for this study (see Fig. 1) incorporates several variables, including elderly service, health status, social adaptation/participation and control factors. Based on their responses to the question, “Did you suffer from hypertension or diabetes diagnosed by physicians,” the participants were divided into two groups, i.e., with and without hypertension/diabetes. Ultimately, the study included 882 elderly migrant participants with hypertension or diabetes and 1266 elderly migrant participants without hypertension and diabetes.

#### Sense of city belonging

A sense of city belonging is the feeling of security and support a member of the city experiences as a result of a sense of acceptance, inclusion, and identity for. The sense of city belonging in this study was measured in 2017 using a five-item scale that consisted of the following items: “I like the city/place where I live now”; “I pay attention to the changes in the city/place where I live”; “I am willing to blend in with the locals and become one of them”; “I feel the locals are willing to accept me as one of them”; and “I feel I am already a local.” Participants were asked the degree to which the statements described their situation with, values ranging from 1 (strongly disagree) to 4 (strongly agree). The Cronbach’s alpha value for the present study was 0.83. We further conducted a confirmatory factor analysis (CFA) to test how well the observed variables represented the latent variable. The GFI, AGFI, and RMSEA values were 1.00 (> 0.90), 0.99 (> 0.90), and 0.042 (≤ 0.05), respectively, thus indicating that the validity of the sense of city belonging scale in this study is good.

#### Elderly service

The study included four predictors of elderly service at the individual level, namely, social security card, medical insurance, convenient access to medical advice, and health education, and two variables at the provincial level, namely, number of licensed doctors per 1000 population and number of hospitals.

##### Social security

To evaluate the social security condition of the MEFF population, this study asked the participants, “Have you ever applied for a social security card?” The respondents were to reply with ‘yes’ or ‘no’.

##### Medical insurance

To measure the degree of acceptance regarding medical insurance, the study asked, “Do you currently have any of the following medical insurance types?” Respondents responded with ‘yes,’ ‘no,’ or ‘unclear.’ The respondents were also asked, “Where did you enroll in your insurance program - the host city, original residence or some other place?” We classified the degree of acceptance of medical insurance into three levels as follows: 0 = not covered by medical insurance or not clear; 1 = participates in a medical insurance program in place of original residence or elsewhere; 2 = participates in a medical insurance program in the local residence.

##### Convenience for seeking medical advice

The transport portability from the place of residence to the nearest medical service institution was used to measure access to convenient medical care. The values ranged from 0 to 3 (0 = more than 1 h; 1 = 30 ~ 60 min; 2 = 15 ~ 30 min; 3 = within 15 min).

##### Health education

To measure health education, this study asked the participants, “Have you obtained information regarding the following health knowledge/health education topics in your current residential community or residential village?” The values ranged from 0 to 1 (0 = no; 1 = yes). We used the number of health education topics for which the participants had received information to evaluate the health education service utilization among the MEFF population.

##### Health resources at provincial level

Licensed doctors are medical workers who have obtained licenses that certify them as doctors and who are employed in medical treatment, disease prevention or healthcare institutions. Hospitals in each province include general hospitals, hospitals specialized in traditional Chinese medicine, hospitals of integrated traditional Chinese and western medicine, ethnic hospitals, specialized hospitals and nursing hospitals.

#### Health status

Self-rated health has been validated as a good indicator of overall health [50]. We used self-rated health to evaluate the participants’ health status. Self-rated health for this study was assessed by asking the respondents, “What is your health status?” The respondents self-rated their health status using four options: 0 = no self-care; 1 = unhealthy, self-care; 2 = fairly healthy; 3 = healthy.

#### Social adaptation

Social adaptation refers to the dynamic relationship between the individual and their social environment [51]. Settlement intention, and local community contact were measured as two dimensions of social adaptation. Regarding settlement intention, participants were asked, “Do you plan to stay here in the future?” The responses ranged from 0 to 3 (0 = no, 1 = uncertain, and 2 = yes). With respect to local community contact, participants were asked, “Do you think you interact well with locals?” The responses ranged from 0 to 1 (0 = no and 1 = yes).

#### Social participation

Social participation was viewed as a lifelong engagement process in which individuals participate in the decision making processes related to the institutions, programs, and environments that affect them [52, 53]. Some scholars equate social participation as involvement in society [54]. Social participation within a community context is described as engaging in a number of social activities in both formal and informal social networks [55]. Social participation in this study was assessed by membership in social organizations and political participation. Regarding organizational participation, the participants were asked, “Which of the following activities have you attended locally since migrating to this city?” The response options were yes or no. With respect to political participation, participants were asked, ““Have you engaged in the following behaviors since migrating to this city?” The respondents answered using a 5-point Likert scale whose values ranged from 1 to 4 as follows: 1 = none, 2 = occasionally; 3 = sometimes; 4 = often).

#### Control factors

Control factors included gender (0 = male, 1 = female), educational level, marital status (1 = married, 0 = single, divorced or widow), migration range and migration duration in the host city. Education level was divided into five groups according years of completion as follows: 0 = primary school or lower, 1 = junior high school, 2 = high school, 3 = college degree, 4 = postgraduate). The values for migration status ranged from 0 to 2 as follows: 0 = intercounty within the city; 1 = interprovincial; 2 = intraprovincial. Migration duration was used to measure the length of time the respondents had lived in their current residence/host city as follows: 0 = within 2 years; 1 = 3 ~ 5 years; 2 = 6 ~ 10 years; 3 = over 10 years).

### Statistical analysis

We used descriptive statistics to understand the demographic characteristics and health status of the participants. The provincial disparities in the distribution of health resources have been a major challenge in China [56]. The cities in China organized by province, administrative region, autonomous region, or municipality. Health systems and medical resources may vary widely between provinces, but may be similar between cities within province. Thus, we also considered the associations of health resources (number of doctors and hospitals) at the province level on sense of city belonging among the MEFFs.

The hierarchical linear modeling (HLM) allows the specification of complex nested, and is used to analyze variance in the outcome variables when the independent variables are at varying hierarchical levels [57]. In this study, MEFFs (at level 1) are nested within provinces (at level 2). The MEFFs in certain province share variance according to their common province. The allocation of health resources among provinces is unbalanced, and the elderly in the same province may belonging to similar system and enjoy similar health resources. We cannot ignore the dependence of each individual in level 1, which violates the independence condition of the Ordinary Least Squares (OLS) model. This study analyze elderly services at the individual level and health resources (number of doctors and hospitals) at the province level. There is statistical ecological fallacy according to interpreting of variable level. HLM is decrease the misinterpreting of analysing outcomes. The MEFFs come from 31 provinces in China, and 30 is the acceptable number of groups [58]. Thus, we further conducted HLM to analyze the effects of individual and provincial elderly services on the sense of city belonging among the MEFFs with and without hypertension/diabetes. Specifically, a two-level HLM model was used for the analysis, in which Level-1 variables were individual MEFFs and Level-2 variables were provinces.

Statistical analysis was performed using SAS 9.4 (Copyright © SAS Institute Inc., SAS Campus Drive, Cary, North Carolina 27,513, USA. All rights reserved.). This study used HLM 7.0 (Copyright ©2011 By Scientific Software International, Inc. All rights reserved.) to conduct multilevel analysis, and LISREL 8.80 was used to conduct the confirmatory factor analysis (CFA).

## Results

### Migration reasons of elderly migrants

**Fig. 2:**
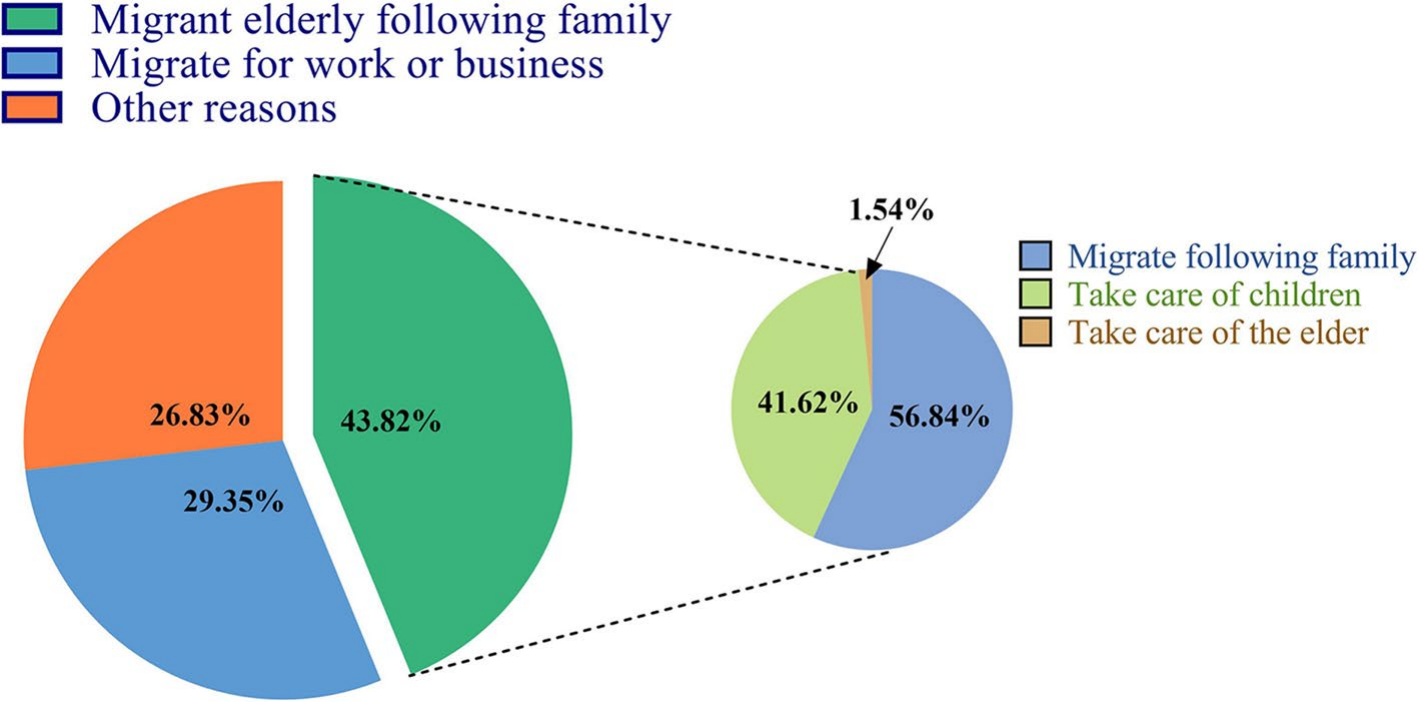
Migration reasons of the elderly migrants aged 60 years old and older in 2017 China Migrants Dynamic Survey

Approximately 43.92% of the elderly migrants in the 2017 China Migrants Dynamic Survey migrated following their families (Fig. 2). In addition, the majority of the MEFFs migrated for the purpose of reuniting with their families (56.84%, 1221), followed by caring for the younger generations (41.62%, 894).

### Sample characteristics of the MEFF

Table 1 presents the results of the descriptive statistics. The proportion of MEFFs living with hypertension/diabetes was 41.06%. The majority of the MEFFs were female. Most of the participants had only primary school or less, and approximately 82.43% of the MEFFs with hypertension or diabetes and 83.10% of the MEFFs without hypertension and diabetes were married. The proportions of the three migration range groups (intercounty within the city, interprovincial, intraprovincial) among the MEFFs with hypertension or diabetes were 18.48, 32.31, and 49.21%, respectively, and the proportions among the MEFFs without hypertension and diabetes were 17.61, 33.25, and 49.13%, respectively. Furthermore, approximately half of the MEFFs lived in their current residences for more than ten years (54.42% of the MEFFs with hypertension or diabetes, 46.13% of the MEFFs without hypertension and diabetes). Only 25.96% of the MEFFs with hypertension or diabetes rated themselves as healthy.

**Table 1:** Sample characteristics of the migrant elderly following family

Characteristics		Total (*n* = 2148)	Participants with Hypertension or Diabetes (*n* = 882)	Participants without Hypertension and Diabetes (*n* = 1266)
**Gender**				
Male		946 (44.04)	395 (44.78)	551 (43.52)
Female		1202 (55.96)	487 (55.22)	715 (56.48)
**Marital Status**				
Married		369 (17.18)	155 (17.57)	214 (16.90)
Single		1779 (82.82)	727 (82.43)	1052 (83.10)
**Education Level**				
Primary School or Lower		946 (44.04)	398 (45.12)	548 (43.29)
Junior High School		619 (28.82)	253 (28.68)	366 (28.91)
High School		382 (17.78)	141 (15.99)	241 (19.04)
College Degree		199 (9.26)	90 (10.20)	109 (8.61)
Postgraduate		2 (0.09)	0 (0)	2 (0.16)
**Migration Range**				
Intercounty within the city		386 (17.97)	163 (18.48)	223 (17.61)
Interprovincial		706 (32.87)	285 (32.31)	421 (33.25)
Intraprovincial		1056 (49.16)	434 (49.21)	622 (49.13)
**Migration Duration**				
Within 2 Years		325 (15.13)	105 (11.90)	220 (17.38)
3 ~ 5 Years		290 (13.50)	111 (12.59)	179 (14.14)
6 ~ 10 Years		469 (21.83)	186 (21.09)	283 (22.35)
Over 10 Years		1064 (49.53)	480 (54.42)	584 (46.13)
**Self-Rated Health Status**				
No Self-Care		29 (1.35)	16 (1.81)	13 (1.03)
Unhealthy, Self-Care		374 (17.41)	215 (24.38)	159 (12.56)
Fairly Healthy		900 (41.90)	422 (47.85)	478 (37.76)
Healthy		845 (39.34)	229 (25.96)	616 (48.66)

### Sense of city belonging and elderly service utilization by the MEFFs

The mean reported sense of city belonging of the MEFF (on a scale of 5–20) was 17.25, with the MEFF scores with and without hypertension and diabetes were 17.28 (SD 2.40) and 17.23 (SD 2.47), respectively. Among the MEFFs with hypertension or diabetes and the MEFFs without hypertension or diabetes, the majority had a social security card (60.66%, 882; 59.87%, 758), lived less than 15 min from the nearest medical service institution (73.58%, 649; 76.38%, 967), and had received education on an average of more than two health education topics. Only 10.54% of the MEFFs with hypertension or diabetes and 8.06% of the MEFFs without hypertension and diabetes participated in medical insurance in their local place of residence.

### The effect of elderly services on sense of city belonging among the MEFFs with and without hypertension and diabetes

Table 2 shows the results of hierarchical linear modeling. The results of model 1 indicate the impact of individual and provincial elderly services on sense of city belonging among the MEFFs with hypertension or diabetes, accounting for control factors at the individual level. The variance between-group is significant (χ^2^ = 66.32, *p* < 0.001). At the individual level, receiving health education was positively associated with a sense of city belonging (γ = 0.05, *p* = 0.033), while owning a social security card, enrolling in medical insurance, and having access to the convenience of medical advice exhibited no significant effect on the sense of city belonging among MEFFs with hypertension or diabetes (*p* > 0.05). In addition, more licensed doctors per 1000 population (γ = 0.39, *p* < 0.001) and the number of hospitals (γ = 0.00, *p* < 0.042) at the provincial level were found to enhance individual sense of city belonging.

Model 2 indicates the effect of individual and provincial elderly services on the sense of city belonging among migrants who had been diagnosed with hypertension or diabetes. The variance between-group is significant (χ^2^ = 51.90, *p* = 0.008). The results indicate they may have a greater sense of city belonging when they own a social security card (γ = 0.57, *p* < 0.001) and receive health education on a wider range of topics (γ = 0.07, *p* = 0.018). Participation in medical insurance reduced the sense of city belonging among the MEFFs without hypertension and diabetes (*p* < 0.01).

**Table 2:** The effects of individual and provincial factors on sense of city belonging among the migrant elderly following family with and without hypertension and diabetes

	Model 1		Model 2	
	γ	p	γ	p
Fixed effect				
Intercept (γ00)	16.36	< 0.001^***^	17.14	< 0.001^***^
**<Provincial level>**				
Licensed doctors per 1000 population	0.39	< 0.001^***^	-0.12	0.069
Number of hospitals	0.00	0.042^*^	0.00	0.987
**<Individual level>**				
** Elderly services**				
Social security card	0.18	0.211	0.57	< 0.001^***^
Medical insurance (participant in original residence or elsewhere vs. no)	-0.34	0.218	-0.79	< 0.001^***^
Medical insurance (participant in local residence vs. no)	-0.26	0.392	-1.01	0.005^**^
Transportation convenience to hospitals	0.08	0.663	0.22	0.125
Health education	0.05	0.033^*^	0.07	0.018^*^
** Health status**				
Self-rated health	0.05	0.600	0.10	0.324
** Social adaptation**				
Settlement intention (uncertain vs. no)	0.09	0.477	0.09	0.689
Settlement intention (yes vs. no)	1.38	< 0.001^***^	1.00	< 0.001^***^
Local community contact	0.42	0.026^*^	0.39	0.017^*^
** Social participation**				
Organization participation	0.18	0.239	0.09	0.225
Political participation	0.08	0.079	0.08	0.079

The results of model 1 show the individual and provincial factors on sense of city belonging among the participants with hypertension or diabetes, while model 2 presents the hierarchical linear modelling results among the participants without hypertension and diabetes

### The effects of social adaptation on sense of city belonging

In addition, the MEFFs who interacted with locals had a better sense of city belonging (*p* < 0.05), and the MEFFs who were willing to remain in the current city in the future exhibited a higher sense of city belonging than those who did not plan to remain (*p* < 0.05).

## Discussion

This study analyzed how to improve he MEFFs’ sense of city belonging from an elderly services perspective using hierarchical linear modeling. This research provides important information for elderly care planners and public health policy makers. We should take different strategies from the elderly service perspective to upgrade sense of city belonging among the MEFF with or without chronic disease status, such as hypertension or diabetes. Furthermore, for MEFFs with hypertension or diabetes, health managers should focus on improving inclusive elderly services that incorporate health education and increase the number of doctors per 1000 population and the number of hospitals to enhance the sense of city belonging. The government should strengthen social security and health education so as to facilitate better adaptation and integration of the MEFFs without hypertension or diabetes into the host city.

Meeting the demand for health education may enhance the sense of city belonging among the MEFF population. Some scholars have indicated that health education programs promote a greater sense of city belonging [59, 60]. This study also found that delivering a wider range of health knowledge and information to the MEFFs has a significant positive effect on migrants’ sense of city belonging. This prompts us to strengthen broader government-led or whole community prevention interventions and to involve communication practitioners to increase health literacy with the aim of reducing the risk factors for illness through targeted health education.

Access to health care is interwoven with questions of non/belonging [61]. Elderly migrants who joined social medical insurance programs experienced greater social integration as applying for social security cards increased their sense of belonging among internal migrants [62, 63]. Our results also found that the MEFFs without hypertension or diabetes who owned a social security card experienced a greater sense of city belonging. Elderly migrants with social security cards enjoyed preferential services for seniors such as pension issuance, transportation access, discounts on tickets for some cultural and entertainment venues, etc. Thus, it is suggested that access to social security coverage be increased as a means to further improve the sense of city belonging among the MEFF population who are not diagnosed with hypertension or diabetes.

Enrolling in medical insurance was not significantly associated with a sense of city belonging among the MEFFs with chronic diseases in this study. In fact, the MEFFs without hypertension or diabetes exhibited little sense of city belonging even when enrolled in medical insurance. However, Previous study found that health insurance could improve psychological identity among migrants [63, 64]. The reason for the inconsistency between our results and those of other scholars may be related to the different research participants. Most of the MEFF migrated to the cities to unite with their families, support their children’s careers and care for their grandchildren. Their lifestyle, social welfare, and public health resource utilization may well differ from those of people who migrate for work. The MEFF may experience the double torture of social alienation and economic difficulty. For instance, paying medical insurance would increase their financial pressures, especially when they do not have chronic diseases. In addition, there may be gaps among the nominal reimbursement ratio, the actual reimbursement ratio and the out-of-pocket ratio due to migration [65]. This study indicates that the elderly migrants’ actual needs and expectations regarding medical resources should be considered during the development of elderly service programs in appropriately concordant cities.

Improving medical resource allocation at the provincial level may enhance the sense of city belonging among the MEFFs who are living with chronic diseases. This study indicates that the MEFFs living with hypertension or diabetes may have a better sense of city belonging when they are in those regions with a greater number of licensed doctors per 1000 population and a greater number of hospitals compared to those provinces with fewer medical staff and hospitals. The current medical care practitioners are often transferred from previous fields of practice, such as geriatric medicine and elderly care. Accordingly, the scale and capacity of elderly service practitioners must be continuously improved and the practitioners must be given additional professional training that focuses on elderly services for the MEFF population.

This study indicates that the improvement of elderly services, including health education and the number of licensed doctors and hospitals, contributes to enhancing the sense of city belonging among the MEFFs with chronic diseases. To implement an elderly care system that combines medical care and health care, some areas implemented the three masters co-management model, which is a healthcare team consisting of a specialist from a tertiary hospital, a general practitioner from a community health center, and a certified health manager trained to provide customized and continuous intervention for chronic disease patients. Some studies have verified the effects of the three divisions of co-management, which focuses on self-management, health literacy improvement, and control of chronic disease progression [66–68]. We recommend that health agencies take effective measures to enhance medical staff and improve health management for MEFFs with chronic diseases, and thereby enhance the sense of city belonging among the MEFF population.

In addition, we found that better social adaptation significantly increased the sense of belonging to the city for the MEFF population, as participating in neighborhood affairs and neighborhood social activities may help migrants establish a host city identity [19]. The government’s settlement programs also play an important role in facilitating social integration among migrants [69]. Accordingly, to improve the sense of city belonging among the MEFFs, this study highlights the importance of allowing elderly migrants to settle with their families, and it further recommends improving the age-friendly social environments with respect by establishing policies and promoting preferential friendly relationships between the locals and the migrants.

The results of the present study have several practical implications. The positive relationships between health resources and sense of city belonging among MEFFs with hypertension and diabetes indicated that public policymakers should improve medical supply, including number of doctors and hospitals, and access to health education. Hierarchical diagnosis and treatment policy (HDTP) is the key to improving both efficiency and quality of the medical service system, and solving the problem of insufficient and unbalanced medical resources [70, 71]. Elderly migrants suffer from more chronic diseases may face the problem of the difficulty in seeing a doctor. A practical implication of this study is to accelerate the development of the mode of the chronic disease first and the co-management of doctors in three medical institutions levels. Health education professionals are encouraged to strengthen the health promotion actions for the MEFFs. However, to promote sense of city belonging of the MEFFs without chronic diseases, it is necessary to focus more on improving their social security rather than medical resources. With the transformation of social economy and the increase in the number of the MEFFs, this study highlights that beneficial and acceptable social and health care systems should be developed for the MEFFs without hypertension and diabetes. Other potential applied value derives from the finding that local community contact was positively related with sense of city belonging of the MEFFs. This finding suggests that social works could create age-friendly spaces and organize some entertainment activities or mental health promotion interventions for the seniors to enhance communication and integration between the MEFFs and local residents.

### Limitations

Our data, which were based on nationally representative samples in China, add public health novels about how to improve the sense of city belonging among the MEFF population. However, this study has several limitations. First, the cross-sectional design of the study limits any causal inferences among the variables of interest. Prospective cohort study designs using elderly migrants following their families and panel data analyses will be needed in future research. Second, the mechanism of these independent variables impacting the sense of city belonging among MEFFs requires further research. Third, there is no unified indicator to assess the sense of city belonging. The five questions used in this study to assess the sense of city belonging may have biased the reliability and validity of the results. Finally, this study applied a two-level HLM to analyze associations of elderly services and sense of city belonging for MEFFs. The dependent variable is sense of city belonging, while the level 2 were provincial variables, which may reduce the accuracy of the estimates. However, the provincial data were derived from China National Statistical Yearbooks in 2017, which was objective and could minimize aggregation errors. Other researchers could collect representative sample of the MEFFs at city and province levels, and further explore the effect of elderly services on their sense of city belonging.

## Conclusion

The elderly migrants may face challenges of city integration and sense of belonging due to migrating to new communities. This study analysed appropriate strategies to improve the sense of belonging among the MEFFs from elderly services perspective. For the better development of the city and to foster an aging-friendly social environment, there should be a focus on national undertakings for the aged and minimizing or even eliminating the inequality in elderly services for the MEFF. Specifically, health managers must increase the number of doctors and hospitals at the provincial level and improve health education so MEFFs with chronic diseases will experience a greater sense of belonging to the host city. Public policy should develop elderly services based on the needs of the MEFFs without hypertension and diabetes, such as providing social security cards and health education. We also suggest encouraging MEFFs to positively adapt to the host city, thereby improving their sense of city belonging.

## Data Availability

The datasets generated and/or analysed during the current study are available in the [Migrant Population Service Center, National Health Commission P.R.China] repository, https://www.chinaldrk.org.cn/wjw/#/home]; the [National Bureau of Statistics of China] repository, [http://www.stats.gov.cn/tjsj/ndsj/2017/indexch.htm].
